# *Panax quinquefolium saponin* decreases atherosclerosis in ovariectomized ApoE^−/−^ mice via regulating estrogen receptor α

**DOI:** 10.1186/s13020-026-01410-3

**Published:** 2026-06-22

**Authors:** Li Chen, Jun-He Shi, Xiao-Juan Ma, Hua Chai, Kang-Kang Wei, Zhen Yang, Yu Tan, Hong-Bo Huang, Da-Zhuo Shi, Hua Qu

**Affiliations:** 1 Xiyuan Hospital, China Academy of Chinese Medical Sciences, Beijing, Beijing, 100091 China; 2 National Clinical Research Center for Chinese Medicine Cardiology, Beijing, Beijing, 100091 China; 3 NMPA Key Laboratory for Clinical Research and Evaluation of Traditional Chinese Medicine, Beijing, Beijing, 100091 China; 4 Hepingli Hospital, Beijing, Beijing, 100013 China; 5 Xiyuan Hospital, National Clinical Research Center for Chinese Medicine Cardiology, China Academy of Chinese Medical Sciences, No. 1 Xinyuan Caochang, Haidian District, Beijing, 100091 China

**Keywords:** Panax quinquefolium saponin, Ovariectomized ApoE^−/−^ mice, Estrogen receptor alpha, Atherosclerosis, Inflammation, Apoptosis

## Abstract

**Background:**

Postmenopausal women are at an increased risk for atherogenesis. Panax quinquefolium saponin (PQS) has demonstrated beneficial effects on decreasing inflammatory response, alleviating oxidative stress and improving endothelial function. However, the effect of PQS on atherogenesis in postmenopausal women remains unknown. This study explored the effects of PQS on different pathological stages (8 or 14 weeks) of atherogenesis in ApoE^−/−^ mice with mimicking postmenopausal condition by ovariectomy.

**Methods:**

Atherosclerotic plaque, levels of serum inflammatory cytokines and apoptosis of aortic endothelium were detected. Human umbilical vein endothelial cells (HUVECs) treated with oxidized low-density lipoprotein (ox-LDL) were used to investigate the mechanism. Using network pharmacology, molecular docking and receptor inhibitor to explore the target of PQS in ovariectomized ApoE^−/−^ mice.

**Results:**

ApoE^−/−^ mice subjected to ovariectomy displayed an increase in atherosclerotic plaque formation, levels of serum inflammatory cytokines and apoptosis of aortic endothelial cells. PQS treatment counteracted these detrimental effects both at 8 and 14 weeks. Network pharmacology and molecular docking analysis revealed that PQS targeted estrogen receptor α (ERα) in ovariectomized ApoE^−/−^ mice, a key regulator in inflammation and aortic endothelial apoptosis. PQS regulated inflammation and apoptosis via ERα/PI3K/Akt and ERα/MEK/ERK1/2 pathways. ERα inhibitor mostly counteracted the beneficial effects of PQS on ovariectomized ApoE^−/−^ mice and injured HUVECs, in contrast, ERα activator enhanced these effects.

**Conclusions:**

The study indicated that PQS decreased atherosclerotic plaque by reducing inflammation and apoptosis of aortic endothelium in ovariectomized ApoE^−/−^ mice via regulating ERα, providing new insights into PQS as a therapeutic target in postmenopausal atherosclerosis.

**Graphical abstract:**

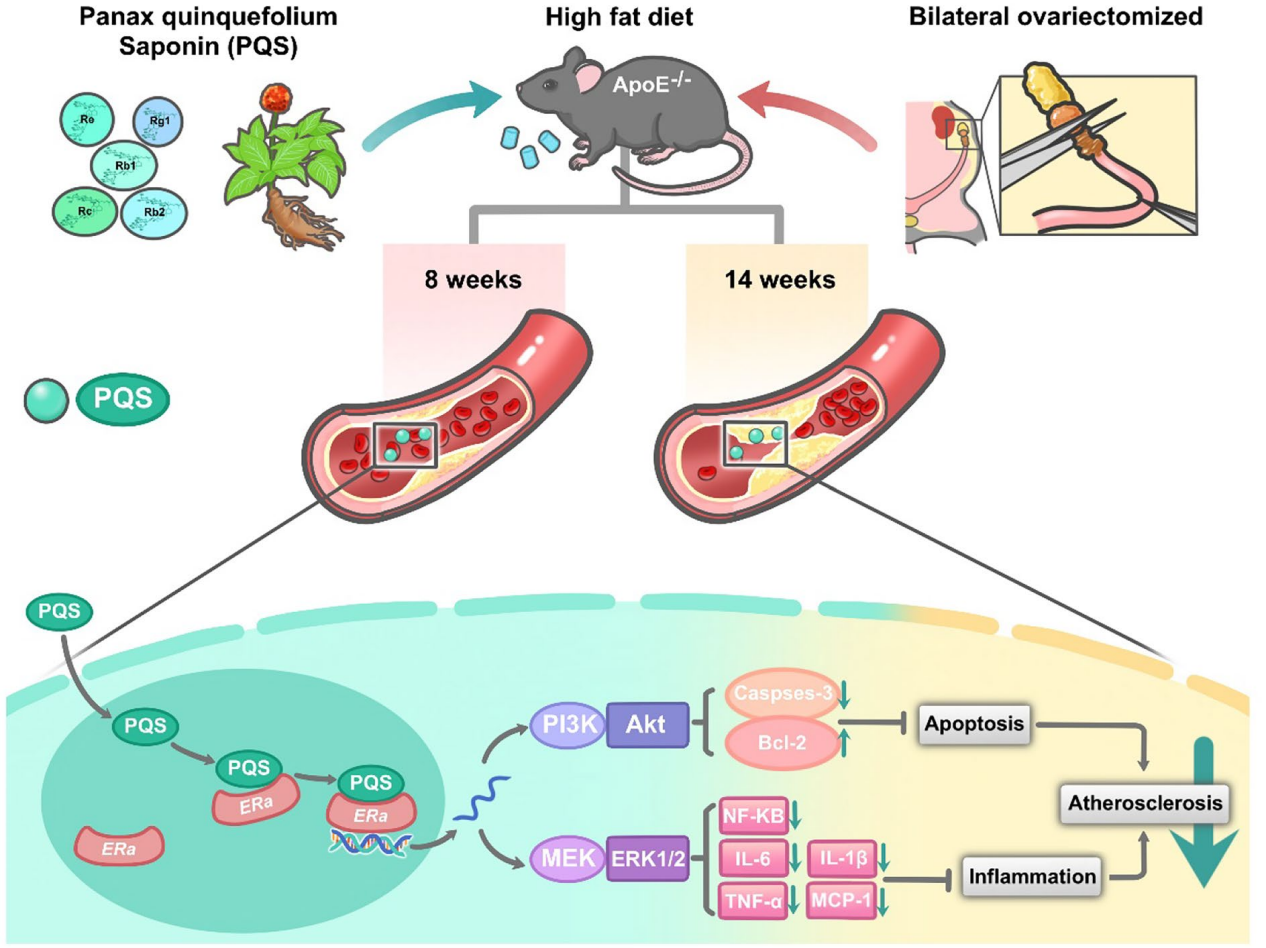

**Supplementary Information:**

The online version contains supplementary material available at 10.1186/s13020-026-01410-3.

## Highlights

Panax quinquefolium saponin (PQS) reduced atherosclerotic plaque, levels of serum inflammatory cytokines and apoptosis of aortic endothelium in ovariectomized ApoE^-/-^ mice at both 8 and 14 weeks, whereas estrogen (E2) treatment only showed a reduction in plaque at 8 weeks. The anti-atherosclerotic effects of PQS in OVX ApoE^-/-^ mice were associated with regulating ERα, a key regulator in inflammation response and aortic endothelial apoptosis.

**Supplementary Information:**

The online version contains supplementary material available at 10.1186/s13020-026-01410-3.

## Introduction

During the period of menopause, estrogen levels rapidly decline, significantly increasing the risk of atherosclerotic cardiovascular diseases (ACVD) [1]. Estrogen exerts its effects by binding to the estrogen receptor alpha (ERα) in the cytosol [2]. In order to dimerize and transfer to the nucleus, the estrogen-bound ERα changes its conformation and then regulates gene transcription by combining with DNA sequences (named estrogen response elements) and other transcription factors [2, 3]. The reduction of ERα is closely associated with increased inflammation response and endothelial cells apoptosis in menopause women [2, 3]. In the postmenopausal phase, activated ERα enhances plaque stability via inhibitory effects on proinflammatory mechanisms [4]. Emerging evidences suggest that the deficiency of endogenous estrogen triggers the acceleration of inflammation and apoptosis in endothelial cells via diminishing the expression of ERα, subsequently inhibited NF-κB, p53 and mitigated mitochondrial redox response [5, 6], which may serve as a catalyst in the development of atherosclerosis in postmenopausal women [7–9].

Hormone replacement therapy (HRT) has been controversial over the past decades. Some studies showed certain benefits, including a reduction in morbidity and mortality from ACVD [10–12]; in contrast, several randomized trials, such as the Women's Health Initiative (WHI), indicated a higher risk of ACVD associated with HRT [13–15]. Pooling recent studies, HRT appears to yield favorable outcomes in women aged 50–60, without increasing risks of cardiovascular events. However, in women over the age of 65, HRT is associated with elevated risks of atherosclerosis and breast cancer [15–17]. Considering the side effects of HRT, a growing number of traditional Chinese medicines have shown significant efficacy in treating menopausal syndrome in women, with no apparent toxic or adverse effects.

Based on traditional Chinese medicine theory, a syndrome combining Qi-Yin deficiency and blood stasis is frequently observed in coronary heart disease (CHD) [18]. The therapeutic use of American ginseng (*Panax quinquefolius L*.; *Araliaceae*) in CHD is ascribed to its properties of tonifying qi and yin [19]. The principal active extract of this plant, *Panax Quinquefolium Saponins* (PQS), is formulated as Xinyue capsule (approval number: Z20030073)—a Chinese herbal patent medicine that has been utilized for more than 20 years. Our previous randomized, double-blind clinical trial demonstrated that PQS decreased the risk of cardiovascular events following percutaneous coronary intervention (PCI) in patients with stable CHD [20]. However, the effect of PQS on atherogenesis in postmenopausal women remains unknown. Therefore, in the present study, both ovariectomized (OVX) ApoE^−/−^ mice feeding with high-fat diet (HFD) and injured human umbilical vein endothelial cells (HUVECs) induced by oxidized low-density lipoprotein (ox-LDL) were applied to explore the effects of PQS on atherogenesis mimicking the physiopathologic condition in postmenopausal women and to explore its potential underlying mechanisms.

## Materials and methods

### Reagents

PQS was acquired from Yisheng Pharmaceutical Co., Ltd. (Jilin, China; Batch No. N121114), dissolved in high-pressure sterilization water and kept at 4 °C. Estradiol valerate tablet (E2) were bought from Bayer HealthCare Co., Ltd (Guangzhou, China, Lot: J20171038), dissolved in high-pressure sterilization water and kept at 4 °C. Endothelial cell medium (ECM) was bought from ScienCell Research Laboratories (Carlsbad, USA, Lot: #1001). Ox-LDL (purity > 97%) was purchased from Peking Union-Biology Co. Ltd (Beijing, China, Lot: UBC-ox-LDL5). β–estradiol (purity ≥ 98%) and PPT (1,3,5-Tris(4-hydroxyphenyl)−4-propyl-1H-pyrazole, purity ≥ 98%) were bought from Sigma–Aldrich (Saint Louis, USA, Lot: E2758, H6036). β–estradiol was lysed in anhydrous alcohol and kept at − 20 °C. ICI 182780 (purity ≥ 99%) was bought from MedChemExpress Co, Ltd (Shanghai, China, Lot: HY-13636). PPT and ICI were lysed in dimethyl sulfoxide and kept at − 20 °C. CCK-8 assay kits were bought from Dojindo Molecular Technologies (Japan, Lot: CK04). Mouse interleukine-6 (IL-6) and IL-1β ELISA kits were bought from Nanjing Jiancheng Bioengineering Institute (Nanjing, China, Lot: 20210326, 20210508). Mouse tumor necrosis factor-α (TNF-α) and monocyte chemoattractant protein-1 (MCP-1) ELISA kits were bought from Cohesion Biosciences (London, UK, Lot: CEK1565, CEK1524). Human IL-6 and TNF-α ELISA kits were bought from multi science (China, Lot: EK106HS-96, EK182HS-96). Human IL-1β and MCP-1 ELISA kits were bought from Bioss Antibodies (China, Lot: bsk11001, bsk11040). FITC Annexin V Apoptosis Detection Kit was bought from BD Biosciences Pharmingen (San Diego, US, Lot: 556547). TRIzol reagent was bought from Invitrogen (Sigma, USA, Lot: 15596018). Antibody for PI3K (#ab40755), Bcl-2 (#ab7973), caspase-3 (#ab184787) was obtained from Abcam (Cambridge, UK). Antibody for ERα (#sc-8005) was bought from Santa Cruz (Santa Cruz, USA). Antibody for Akt (#4685S), phospho-Akt (Ser473, #9271), MEK (#9124S), ERK1/2 (#4695S), phospho-NF-κB (Ser536, #3033), and NF-κB (#8242S) were bought from CST (Massachusetts, USA).

### Experimental animals

All animal experiments were authorized by the Ethics Review Committee for Animal Experimentation of Xiyuan Hospital, China Academy of Chinese Medical Sciences (the approval number: 2021XLC022-2) in November 3, 2021. One hundred and eighty ApoE^−/−^ female mice with a C57BL6/J genetic background (weighing 18 ± 2 g, 6–8 weeks) were bought from Beijing Vital River Laboratory Animal Technology Co., Ltd. Before any experimental interventions and grouping, all mice were acclimatized for 1 week.

To ensure baseline homogeneity across all experimental groups, key parameters including body weight and age among the mouse groups were measured and statistically compared at the end of the acclimatization period (designated as baseline). The random allocation resulted in no significant differences in initial body weight (e.g., mean ± SD: HFD: 20.37 ± 1.15g, HFD + OVX: 20.64 ± 1.04g, HFD + OVX + E2: 20.63 ± 1.33g, HFD + OVX + PQS: 20.41 ± 1.01g, HFD + OVX + PQS + ICI: 20.56 ± 1.33g; p > 0.05), age (all mice were 7–9 weeks), thereby validating the comparability of the groups at the study’s inception.

### Atherosclerosis model in ovariectomized ApoE^−/−^ mice fed with HFD

We anesthetized ApoE^−/−^ mice and cut open the skin of middle back layer to remove ovaries. Following the operation procedure, the wounds were sutured. The ovariectomized model was considered successful if the serum estrogen levels in mice were below 2.5 pg/mL after 2 weeks.

ApoE^−/−^ mice were divided into HFD group (without OVX, fed with HFD diet and distilled water), HFD + OVX group (OVX, fed with HFD diet and distilled water), HFD + OVX + E2 group (OVX, fed with HFD diet and E2 0.13 mg/kg/day), HFD + OVX + PQS group (OVX, fed with HFD diet and PQS 45 mg/kg/day), HFD + OVX + PQS + ICI group (OVX, fed with HFD diet, PQS 45 mg/kg/day and ERα inhibitor ICI 182780 5 mg/kg/every 2 days). Every group has 15 ApoE^−/−^ mice and the treatment was scheduled for either 8 or 14 weeks.

### HUVEC

HUVECs were bought from iXCells Biotechnologies (San Diego, CA, USA, Lot: 200042) and cultured in ECM. HUVECs within passages 4–7 were used, and the ECM was changed every 2 days. HUVECs were stimulated with ox-LDL (0, 50, 100, 150, and 200 μg/mL) at 12, 24, 48, or 72 h, and cell viability was assessed to select the appropriate concentration. HUVECs were then treated with PQS (0, 25, 50, 100, 150, or 200 μg/mL) or E2 (0, 10, 20, 50, 100, or 200 nmol/L) for 24 h to evaluate cell viability and select fitting concentration.

To elucidate the effects of PQS on ox-LDL-induced HUVEC injury, HUVECs were allocated to control group (routine culture for 24 h), ox-LDL group (treating with ox-LDL for 24 h), PQS groups (pretreating with PQS for 2 h, followed by co-treating with ox-LDL for 24 h), E2 group (pretreating with E2 for 2 h, then co-treating with ox-LDL for 24 h). Moreover, we also designed ox-LDL + PQS + ICI group and ox-LDL + PQS + PPT group (pretreating with PQS, ICI or PPT for 2 h and then co-treating with ox-LDL for 24 h) to explore the mechanism of PQS on ox-LDL-stimulated HUVEC injury.

### Cell viability assays

In a 96-well plate, the density of HUVECs was 4000 cells/well. According to above experimental protocol, every well was treated with 10 μL CCK-8 reagent to show the absorbance (OD) value. Then the OD value was set at 450 nm and read by a microplate reader.

### Serum lipids

Samples of blood were obtained from the eyelids and stored at tubes. The blood in tubes was rotated 3000 rpm/min for centrifugation to gather serum. To detect serum lipids levels, we then used TC, TG, HDL-C, and LDL-C measurement kits. Microplate readers were used to determine the OD values.

### Enzyme-linked immunosorbent assay

The levels of IL-6, IL-1β, TNF-α and MCP-1 were measured by ELISA kit. According to instructions, the detecting steps are as follows: (1) standard material was dissolved into 7 distinct concentrations to establish standard solution, (2) putting samples into antibody-shrouded microplates for incubation, (3) washing and then adding streptavidin-HRP working solution, (4) finally adding chromogenic substrate and incubating again, (5) adding stop solution. The levels of IL-6, IL-1β, TNF-α and MCP-1 were calculated based on their corresponding standard curves.

### Oil red O staining

After anesthesia, the aortas were removed and locked in 4% paraformaldehyde. Following fixation, dyeing aortas with a 0.2% Oil Red O staining solution. Washing aortas with PBS to remove excess staining solution. The thoracic aorta and aortic arch were longitudinally cut open and imaged with optical microscope (× 40). The atherosclerotic plaque areas on the thoracic aorta and aortic arch were measured using ImageJ.

### Hematoxylin and eosin (H&E) staining

Using 4% paraformaldehyde to lock aortas. Dehydrating aortas with ethyl alcohol and then infiltrating with xylene, following by embedding in paraffin. The samples were sectioned at 4 μm using a microtome, and tissue-containing microslides were dewaxed and rehydrated. The tissues were dyed with H&E staining kit and finally photographed with optical microscope (× 100).

### TdT-mediated dUTP nick-end labeling (TUNEL)

The previous experimental procedures of the aortas in TUNEL staining were the same as those for H&E staining. The paraffin samples containing aortas were cut into 4-μm pieces. Based on the direction, using In Situ Cell Death Detection Kit (Roche, USA, Lot: 11684795910) to conduct TUNEL staining. The normal cyteblast were light blue, while apoptotic cyteblast were yellow–brown by dyeing. The apoptotic index value was counted by the ratio of apoptotic cells to total cells (× 400).

### Flow cytometry analysis

Applying Annexin V-fluorescein isothiocyanate (A-V-FITC)/propidium iodide (PI) apoptosis detection kit to estimate cells apoptosis. The steps were as follows: (1) collecting and washing cells with 1X binding buffer, (2) labeling cells by adding 5 µl A-V-FITC and 5 µl PI into conjugate buffer, (3) detecting apoptotic cells by employing a flow cytometer, (4) using A-V value to establish abscissa and PI value to establish ordinate, (5) upper left marked mechanically injured cells, upper right marked late apoptotic cells, lower left marked undamaged cells, lower right marked early apoptotic cells in the flow cytometric dot plot.

### Immunofluorescence staining

Harvesting aortas and fixing them in 4% paraformaldehyde, and processed for dual immunofluorescence staining (CD31 and ERα). The aorta tissue sections underwent an overnight incubation at 4 °C with anti-CD31 and anti-ERα primary antibodies, followed by incubating with a biotinylated secondary antibody. Carrying out DAPI dyeing to identify the individual cells. Using a Nikon inverted microscope to capture images. For semiquantitative analysis, both ERα-positive and CD31-positive endothelial cells lining the luminal surface of the aortic sections were determined.

According to the aforementioned protocol. Fixing the cells and washing 3 times, and then incubating overnight with primary antibodies, including ERα, Bcl-2 and spase-3. Washing again and incubating with secondary antibody. Putting 50 μL DAPI solution into the cells for incubation and finally taking pictures with fluorescence microscope (200x) (Observer. Z1, Zeiss, Germany).

### Network pharmacology

Our previously study showed that the main compounds of PQS comprised Ginsenoside Re, Rg1, Rb1, Rc, and Rb2 (Supporting Information 1) [21]. To predict the possible pathway by which PQS influence the levels of estrogen, we input these compounds into BATMAN-TCM [22] and 127 targets were identified using a Perl script, which were shared by five PQS constituents and a BATMAN-TCM score > 3; while for the targets regulating estrogen levels, we obtained 1341 targets from the GeneCards (https://www.genecards.org/) [23], TTD (http://db.idrblab.net/ttd/) [24] and OMIM databases (https://omim.org/) [25]. The overlap targets were then input into the DAVID database to predict pathways and were positioned based on P values [26]. The results of KEGG revealed that the effects of PQS on estrogen levels in postmenopausal women were tightly connected with the ERα/PI3K/Akt signaling pathway.

### Molecular docking

The 2-dimension (2D) structures of Ginsenoside Re, Rg1, Rb1, Rc, and Rb2 in PQS were acquired from the PubChem database [27], and the 3D structure of the ERα was gained from the PDB database [28]. Converting above-mentioned files into PDB format. Resetting ER protein document via using AutoDock4. The following steps demonstrated that shedding water and using hydrogen replacement, as well as assigning the protein as a receptor and saved as PDBQT format. Using AutoDock vina1.1.2 to carry out molecular docking. The best-scoring poses were further analyzed to investigate the interactions between the component of PQS and ERα at the molecular level.

### Real-time PCR (RT–PCR)

Extracting total RNA via applying TRIzol. A 1.5 µM concentration of cDNA was used. Conducting RT–PCR via operating a Bio-Rad MyIQ single-color RT–PCR detection system. For each PCR, two microliters of cDNA were combined with 10 µL of universal PCR master mix, 0.5 µL of forward primer, 0.5 µL of backward primer, and 7 µL of dd H_2_O. PCR amplification of ERα, PI3K, Akt, Bcl-2, caspase-3, MEK, ERK, NF-κB and GAPDH were performed as follows: 1 cycle at 94 °C for 2 min; 45 cycles at 94 °C for 5 s and 60 °C for 30 s; and 1 cycle at 72 °C for 10 min.

A standard curve was conducted to count copy numbers of relative RNA. The expression levels of ERα, PI3K, Akt, Bcl-2, caspase-3, MEK, ERK, NF-κB and GAPDH were counted by the 2^−△△Ct^ method: fold change = 2^−△△Ct^, △△Ct = (Ct _Sample_ − Ct _GAPDH_) − (Ct _Control_ − Ct _GAPDH_). Standardizing expression of target genes by housekeeping gene cyclophilin. The primers in the RT–PCR analysis were displayed in Supplementary Table 1.

### Western blotting

To extract the proteins for each sample in aortic tissue, 30 mg of tissue and 300 µL of lysis buffer were put to a tissue abrader. Then extracting the protein from the HUVECs. The specimens were blended and incubated, following by operating BCA kit (P0012S, Beyotime, Shanghai, China) to calculate protein concentration. The samples received protein loading buffer and were then boiled. According to target proteins' molecular weight, different SDS-PAGE gel concentrations were created, and the sample volume was determined using the detected protein concentration.

Putting specimen into the wells for electrophoresis, and transferring proteins to PVDF membranes. Then blocking the membranes with 5% BSA and being incubated with different antibodies against ERα, PI3K, Akt, P-Akt (Ser473), Bcl-2, caspase-3, MEK, ERK, phospho-NF-κB p65 (Ser536), NF-κB and GAPDH, respectively. Following by being incubated with secondary antibodies. After conducting with ECL luminescent working solution, proteins were developed by gel imaging system, and expression of above proteins was calculated through Image-Pro Plus Analysis Software.

### Statistical analyses

The data are showed with means ± standard deviations (SDs). Analyzing two groups data via t-tests, and more than two groups data via one-way analysis of variance (ANOVA) and the Bonferroni post hoc test. Statistical analyses were conducted by operating Origin2021 software (Origin software, USA). A two-sided P < 0.05 was considered statistically significant.

## Results

### PQS decreases atherosclerotic plaque in ovariectomized ApoE^−/−^ mice

**Fig. 1:**
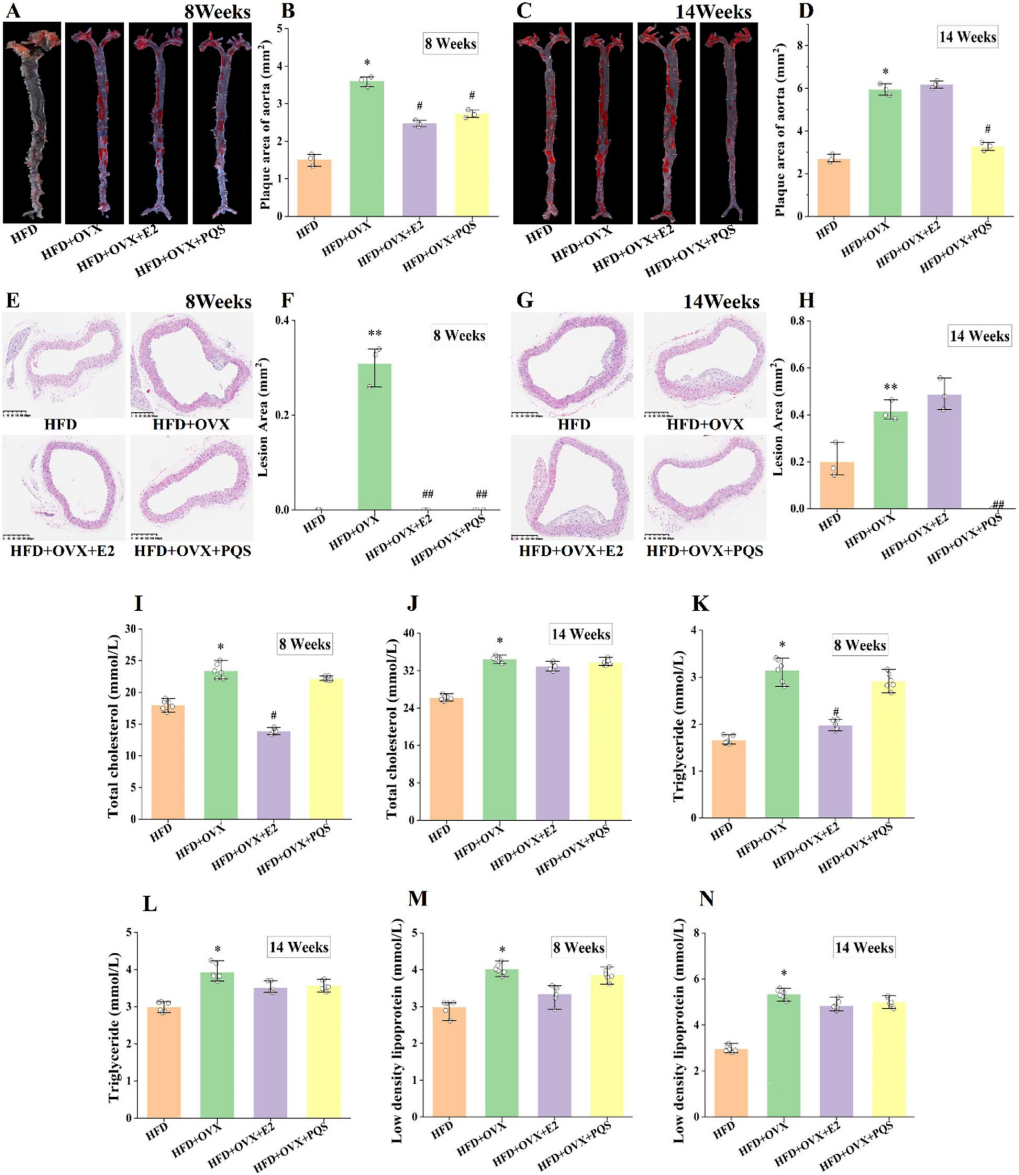
PQS ameliorated atherosclerotic plaque in ovariectomized ApoE^−/−^ mice. **A**–**D** Oil red O staining of aorta and quantitative analysis of atherosclerotic plaque areas at 8 and 14 weeks. **E**–**H** H&E staining of aortic root and quantitative analysis of aortic root atherosclerotic plaque areas at 8 and 14 weeks. **I**–**N** The measurement of serum TC, TG, and LDL-C levels at 8 and 14 weeks. n = 3–6. Data are expressed as the mean ± SEM. ^*^P < 0.05, ^**^P < 0.01 vs. HFD; ^#^P < 0.05, ^##^P < 0.01 vs. HFD + OVX

Histological evaluation of the aortas was conducted to estimate the effects of PQS on atherosclerotic plaques and the results of Oil red O staining and H&E staining showed that ovariectomized ApoE^−/−^ mice showed a pronounced increase in atherosclerotic plaque when compared with ApoE^−/−^ mice without ovariectomy both at 8 and 14 weeks (Fig. 1A–H). PQS treatment reduced atherosclerotic plaque areas in ovariectomized ApoE^−/−^ mice at both 8 and 14 weeks (Fig. 1A–H), while E2 treatment only reduced atherosclerotic plaque areas at 8 weeks (Fig. 1A, B, E, F).

The measurement of blood lipid revealed significant increases in serum TC, TG, and LDL-C levels in ovariectomized ApoE^−/−^ mice when compared with ApoE^−/−^ mice without ovariectomy at 8 and 14 weeks (Fig. 1I–N). PQS had no obvious effects on the levels of TG, TC or LDL-C at either 8 or 14 weeks (Fig. 1I–N). The results indicate that PQS may not be closely associated with lipid profiles in reducing atherosclerotic plaque in ovariectomized ApoE^−/−^ mice.

### PQS decreases the apoptosis of endothelial cells in vivo and vitro

**Fig. 2:**
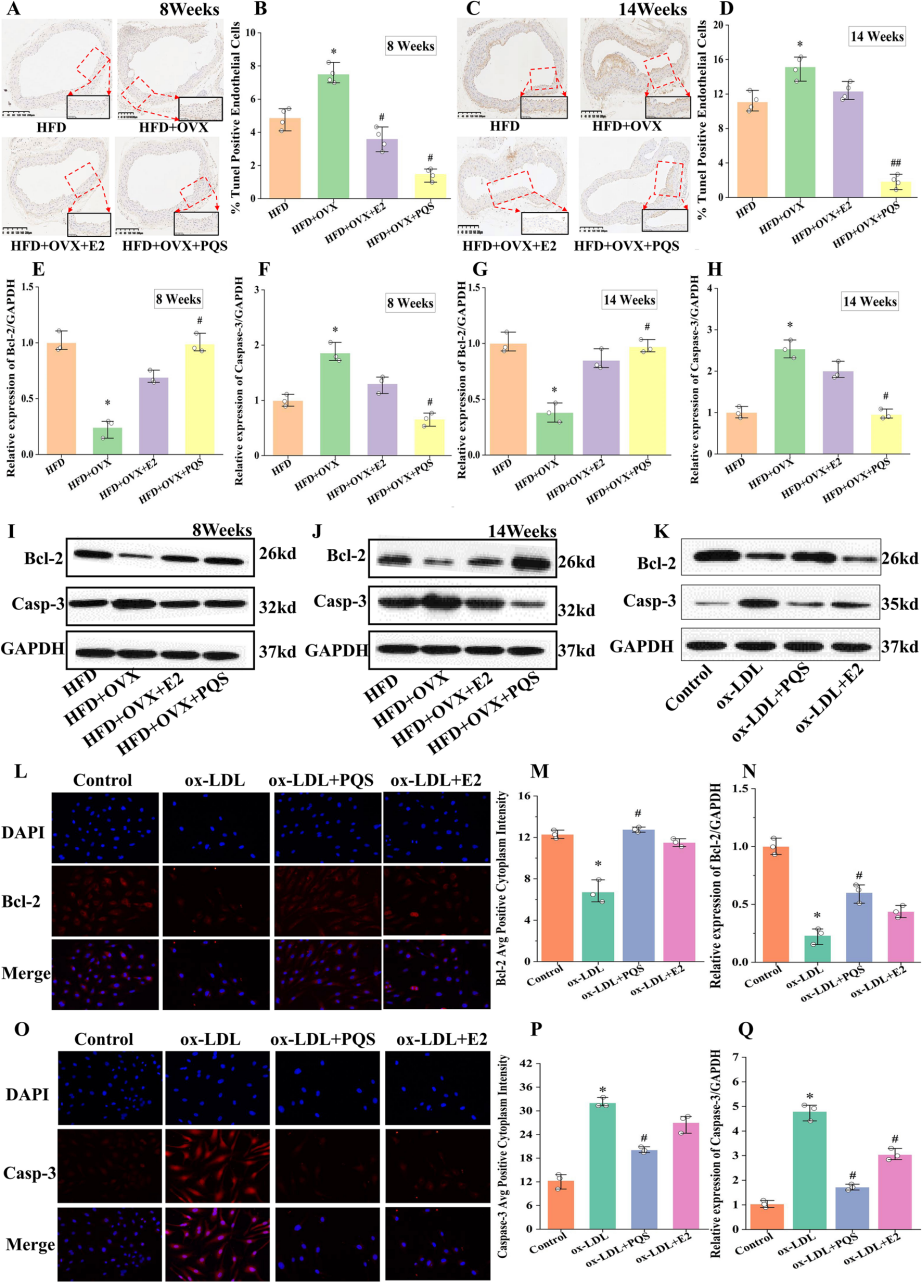
PQS decreased the apoptosis of aortic endothelial cells in vivo and in vitro. **A**–**D** Images and quantitative analysis of aortic TUNEL staining at 8 and 14 weeks. **E**–**J** Images of western blotting and quantitative analysis of Bcl-2 and caspase-3 expression in aortic tissue at 8 and 14 weeks. **K**, **N**, **Q** Images of western blotting and quantitative analysis of Bcl-2 and caspase-3 expression in HUVECs co-treated with PQS and ox-LDL. **L**-**M**, **O**-**P** Immunofluorescence assays (200x) and quantitative analysis of Bcl-2 and caspase-3 in HUVECs co-treated with PQS and ox-LDL. n = 3–6. Data are expressed as the mean ± SEM. ^*^P < 0.05 vs. HFD; ^#^P < 0.05, ^##^P < 0.01 vs. HFD + OVX

During development of atherosclerosis, apoptosis of endothelial cells exacerbates the inflammatory response, coagulation cascade activation, and instability of plaques. We assessed the effects of PQS on apoptosis of endothelial cells and expression of apoptotic proteins in aorta. The apoptosis of endothelial cells in ovariectomized ApoE^−/−^ mice was obviously raised comparing with ApoE^−/−^ mice without ovariectomy, while treating with PQS decreased endothelial cell apoptosis in aortas of ovariectomized ApoE^−/−^ mice at both 8 weeks and 14 weeks (Fig. 2A–D). Moreover, apoptotic signals in aortas of ovariectomized ApoE^−/−^ mice were markedly increased, manifesting diminished Bcl-2 expression and increased pro-caspase-3 expression (Fig. 2E–J, Supplementary Fig. 1A-D). After treating with PQS for 8 weeks or 14 weeks, ovariectomized ApoE^−/−^ mice showed ascended Bcl-2 expression and descended pro-caspase-3 expression in aortas (Fig. 2E–J, Supplementary Fig. 1A-D). Consistent with the reduced apoptosis detected by TUNEL staining, the decreased pro-caspase-3 level suggests a potential anti-apoptotic effect, though cleavage-specific data are needed for confirmation. However, E2 intervention made no effects on caspase-3 expression at either 8 weeks or 14 weeks (Fig. 2F, H, I, J, Supplementary Fig. 1C-D).

We also evaluated the effect of PQS on HUVECs apoptosis stimulated by ox-LDL. The results of flow cytometry assays observed that ox-LDL significantly increased endothelial cell apoptosis and decreased cells migration, while treatment with PQS (50, 100, and 150 µg/mL) reduced cell apoptosis and increased cells migration (Supplementary Fig. 1E-I). Immunofluorescence assays revealed that ox-LDL decreased the Bcl-2 average positive cytoplasm intensity and increased the caspase-3 average positive cytoplasm intensity, while pretreating with PQS increased the Bcl-2 average positive cytoplasm intensity and decreased the caspase-3 average positive cytoplasm intensity (Fig. 2L, M, O, P). Moreover, apoptosis-associated factors, including Bcl-2 and caspase-3, were also assessed. These findings displayed that compared with ox-LDL treatment, PQS treatment enhanced Bcl-2 expression and reduced caspase-3 expression (Fig. 2K, N, Q, Supplementary Fig. 1J-K). These results indicate that the reduction of atherosclerotic plaque by PQS in ovariectomized ApoE^−/−^ mice may be associated with inhibition of apoptosis. Moreover, with prolonged PQS treatment, the anti-apoptotic effect became more pronounced.

### PQS decrease inflammatory response in vivo and vitro

**Fig. 3:**
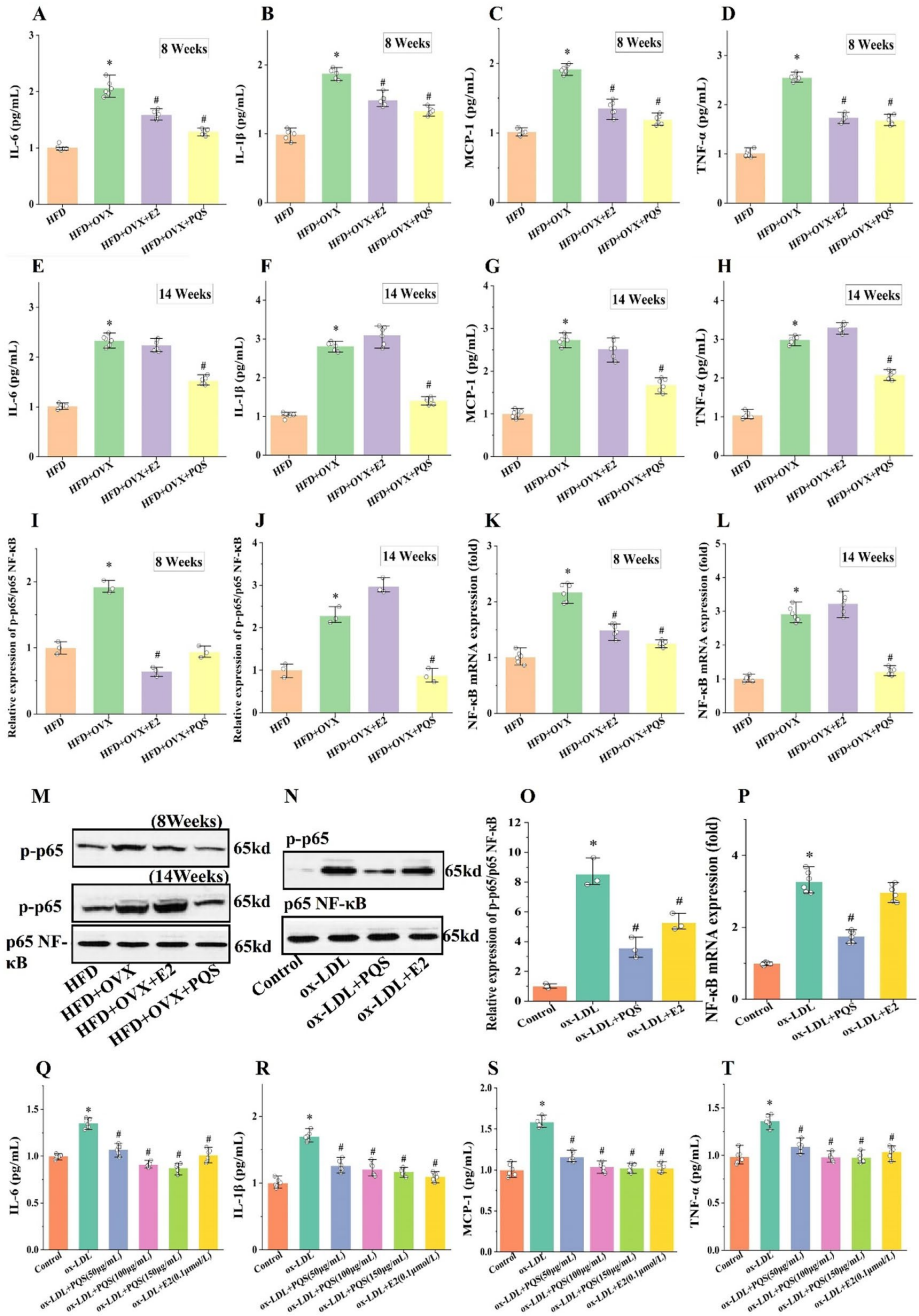
PQS decreased inflammatory response in vivo and vitro. **A**–**H** The levels of serum IL-6, IL-1β, MCP-1 and TNF-α in ovariectomized ApoE^−/−^ mice at 8 and 14 weeks. **I**–**J**, **M-O** Representative western blot images and quantitative analysis of phosphorylated NF-κB p65 (Ser536) relative to total NF-κB p65 in vivo and in vitro. **K**-**L**, **P** mRNA levels of NF-κB in vivo and in vitro. **Q**-**T** The levels of serum IL-6, IL-1β, MCP-1 and TNF-α in HUVECs co-treated with PQS and ox-LDL. n = 3-6. Data are expressed as the mean ± SEM. ^*^P < 0.05 vs. HFD; ^#^P < 0.05 vs. HFD+OVX. ^*^P < 0.05 vs. control; ^#^P < 0.05 vs. ox-LDL

Inflammation is a crucial process that links multiple risk factors for postmenopausal atherosclerosis. To explore the effects of PQS on inflammatory cytokines expression in ovariectomized ApoE^−/−^ mice and ox-LDL-treated HUVECs, we detected the levels of IL-6, IL-1β, TNF-α and MCP-1. The results displayed that the levels of serum IL-6, IL-1β, TNF-α and MCP-1 in ovariectomized ApoE^−/−^ mice were markedly raised at both 8 weeks and 14 weeks compared with those without ovariectomy (Fig. 3A–H). PQS treatment reduced the levels of serum IL-6, IL-1β, TNF-α and MCP-1 at both 8 weeks and 14 weeks (Fig. 3A–H). However, E2 intervention had no obvious effect on the levels of serum IL-6, IL-1β, TNF-α and MCP-1 at 14 weeks (Fig. 3E–H). We also assessed the activation of NF-κB by measuring the ratio of phosphorylated NF-κB p65 (Ser536) to total NF-κB p65, a key indicator of inflammatory pathway activation. We found PQS significantly reduced the p-p65/p65 ratio in ovariectomized ApoE^−/−^ mice at both 8 weeks and 14 weeks (Fig. 3I–M). In vitro studies also showed that compared with ox-LDL treatment alone, PQS treatment decreased the p-p65/p65 ratio (Fig. 3N–P). Moreover, the results in vitro revealed that ox-LDL stimulation evidently enhanced the levels of IL-6, IL-1β, TNF-α and MCP-1 when compared with control group (Fig. 3Q–T). However, treatment with PQS at 50, 100, and 150 µg/mL caused a significant reduction in the levels of IL-6, IL-1β, TNF-α and MCP-1 compared with those in the ox-LDL group (Fig. 3N–Q). These results indicate that PQS decreases atherosclerotic plaque in ovariectomized ApoE^−/−^ mice, and this effect may be associated with inhibiting the inflammatory response.

### PQS influence the activation and expression of ERα in vivo and vitro

**Fig. 4:**
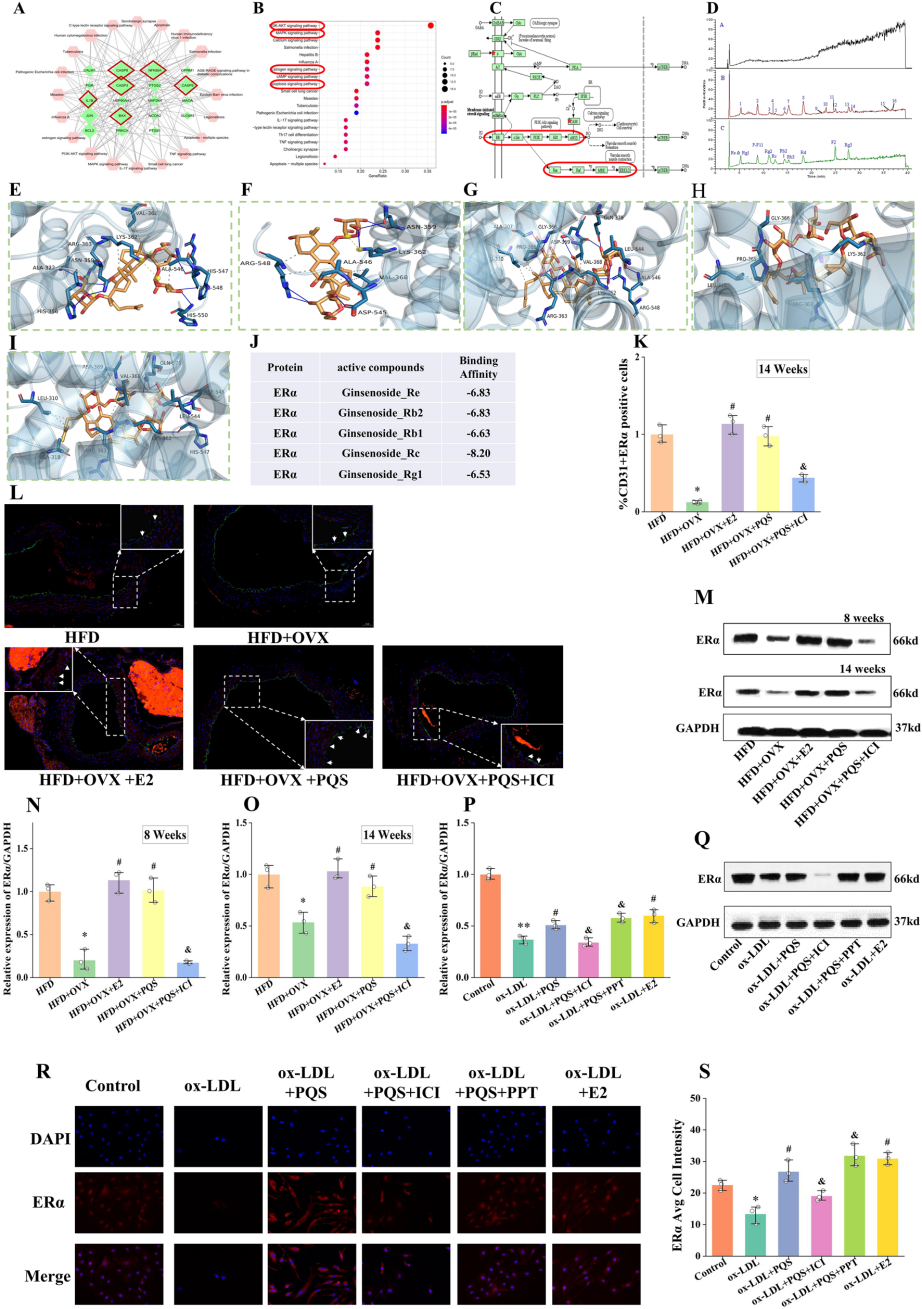
PQS influenced the activation and expression of ERα in vivo and vitro. **A** The top clustered 6 target genes of PQS on atherosclerosis in ovariectomized ApoE^−/−^ mice. **B**-**C** The possible pathway by which PQS influenced atherosclerosis in ovariectomized ApoE^−/−^ mice. **D** Typical base peak chromatograms after injection of PQS. **E**–**J** 3D docking conformation and binding energy of ERα with Ginsenoside Re, Ginsenoside Rg1, Ginsenoside Rb1, Ginsenoside Rc and Ginsenoside Rb2. **K**-**L** CD31 and ERα dual immunofluorescence staining in aortic endothelium and quantitative analysis of CD31 and ERα-positive cells in aortic endothelium at 14 weeks. **M**–**O** Images of western blotting and quantitative analysis of ERα in aortic tissue at 8 and 14 weeks. **P**-**Q** Images of western blotting and quantitative analysis of ERα in HUVECs co-treated with PQS and ox-LDL. **R**-**S** Images of immunofluorescence assays (200x) and quantitative analysis of ERα in HUVECs co-treated with PQS and ox-LDL. n = 3–6. Data are expressed as the mean ± SEM. ^*^P < 0.05 vs. HFD; ^#^P < 0.05 vs. HFD + OVX; ^&^P < 0.05 vs. HFD + OVX + PQS; ^*^P < 0.05, ^**^P < 0.01, vs. control; ^#^P < 0.05 vs. ox-LDL; ^&^P < 0.05 vs. ox-LDL + PQS

Network pharmacology analysis of PQS on atherosclerosis in ovariectomized ApoE^−/−^ mice found that the top clustered 6 target genes included caspase3, NF-κB, caspase8, caspase9, Bax and IL-1β (Fig. 4A), which mainly associated with apoptosis and inflammatory response. The possible pathway by which PQS influenced atherosclerosis in ovariectomized ApoE^−/−^ mice was predicted by KEGG analysis, and we found that the mechanisms were primarily associated with regulating ERα/PI3K/Akt pathway to influence apoptosis and ERα/MEK/ERK1/2 pathway to influence inflammatory response (Fig. 4B, C). Moreover, we conducted molecular docking to verify the binding modes of the 5 main active compounds (Ginsenoside Re, Rg1, Rb1, Rc, and Rb2) of PQS (Fig. 4D) and ERα. The higher absolute value of binding energy, the stronger affinity is shown between Ginsenoside Re, Rg1, Rb1, Rc, Rb2 and ERα (Fig. 4E–J), moreover, the biding affinities ranging from − 6.5 to − 8.8 kcal/mol (Supplementary Table 2). The results demonstrate that Ginsenoside Re, Rg1, Rb1, Rc, and Rb2 have strong affinities with ERα.

We also detected the expression of ERα in aortic tissue, the results showed that ovariectomized ApoE^−/−^ mice exhibited lower expression of ERα in aortic tissue when compared with ApoE^−/−^ mice without ovariectomy, determined by western blotting and RT-PCR (Fig. 4M–O, Supplementary Fig. 2A–B). PQS treatment increased the expression of aortic endothelium ERα in ovariectomized ApoE^−/−^ mice at both 8 and 14 weeks, while inhibiting ERα with ICI 182780 mostly counteracted the effects of PQS on increasing ERα expression (Fig. 4M–O, Supplementary Fig. 2A–B). The results of immunofluorescence staining in the aorta at 14 weeks also demonstrated that PQS treatment increased ERα expression in the aortic endothelium of ovariectomized ApoE^−/−^ mice, while inhibiting ERα counteracted the effects of PQS on increasing ERα expression (Fig. 4K, L). To further explore the effects of PQS on ERα expression, vitro study was performed. The results of western blotting, RT-PCR and immunofluorescence assays also revealed that PQS treatment increased levels of ERα in the injured HUVECs induced by ox-LDL, and ERα inhibitor ICI 182780 mostly offset the effects of PQS on ERα (Fig. 4P–S, Supplementary Fig.  2C).

### Effects of PQS on the downstream pathways of ERα

**Fig. 5:**
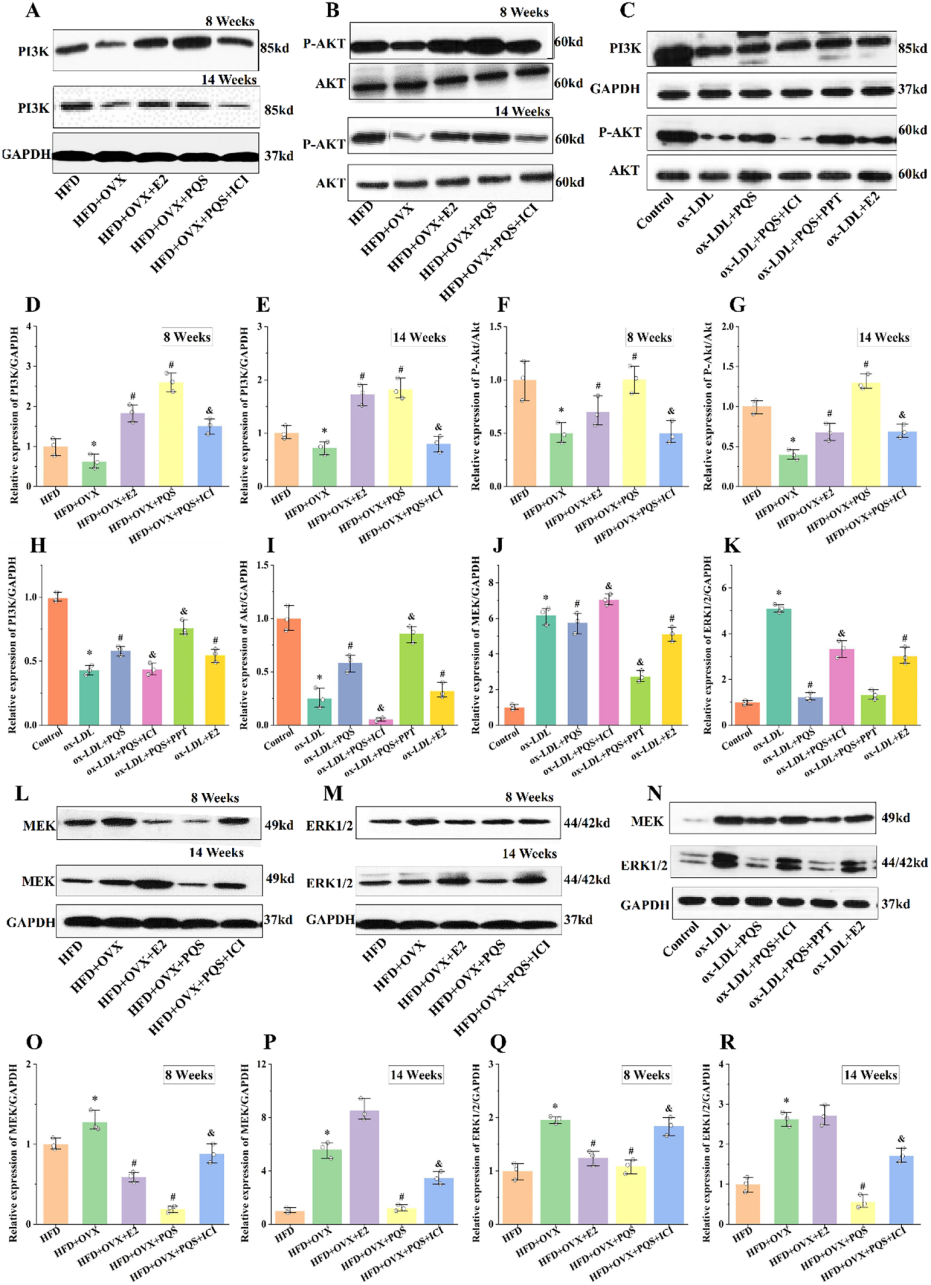
Effect of PQS on ERα/PI3K/Akt and ERα/MEK/ERK1/2 pathway. **A**-**B**, **D**–**G** Images of western blotting and quantitative analysis of PI3K and Akt in aortic tissue at 8 and 14 weeks. **C**, **H**-**I** Images of western blotting and quantitative analysis of PI3K and Akt in HUVECs co-treated with PQS and ox-LDL. **L**-**M**, **O**–**R** Images of western blotting and quantitative analysis of MEK and ERK1/2 in aortic tissue at 8 and 14 weeks. **J**-**K**, **N** Images of western blotting and quantitative analysis of MEK and ERK1/2 in HUVECs co-treated with PQS and ox-LDL. n = 3–6. Data are expressed as the mean ± SEM. ^*^P < 0.05 vs. HFD; ^#^P < 0.05 vs. HFD + OVX; ^&^P < 0.05 vs. HFD + OVX + PQS; ^*^P < 0.05 vs. control; ^#^P < 0.05 vs. ox-LDL; ^&^P < 0.05 vs. ox-LDL + PQS

The effects of PQS on downstream pathways of ERα were also examined in vivo and vitro by western blotting and RT-PCR. The vivo study showed that PQS treatment raised the protein expression of PI3K and the phosphorylated (active) form of Akt (p-Akt). Furthermore, PQS treatment decreased the total protein expression of MEK and ERK1/2 as compared with those in ApoE^−/−^ mice without ovariectomy, while ERα inhibitor ICI 182780 mostly offset the effects of PQS on PI3K, p-Akt, MEK and ERK1/2 (Fig. 5A, B, D–G, L, M, O–R, Supplementary Fig. 3A–H). In vitro study, we also found the similar effects of PQS on PI3K, p-Akt, MEK and ERK1/2 (Fig. 5C, H–K, N, Supplementary Fig. 3I–L). However, ERα inhibitor ICI 182780 mostly offset the effects of PQS on PI3K, p-Akt, MEK and ERK1/2; while ERα activator PPT facilitated these effects (Fig. 5C, H–K, N, Supplementary Fig. 3I–L). These results indicate that PQS increased the expression of PI3K and p-Akt, as well as decreased the expression of MEK and ERK1/2. The effects of PQS on these proteins were largely reversed by an ERα inhibitor, suggesting that ERα may mediate these changes.

### Inhibiting ERα counteracts the beneficial effects of PQS in vivo and vitro

**Fig. 6:**
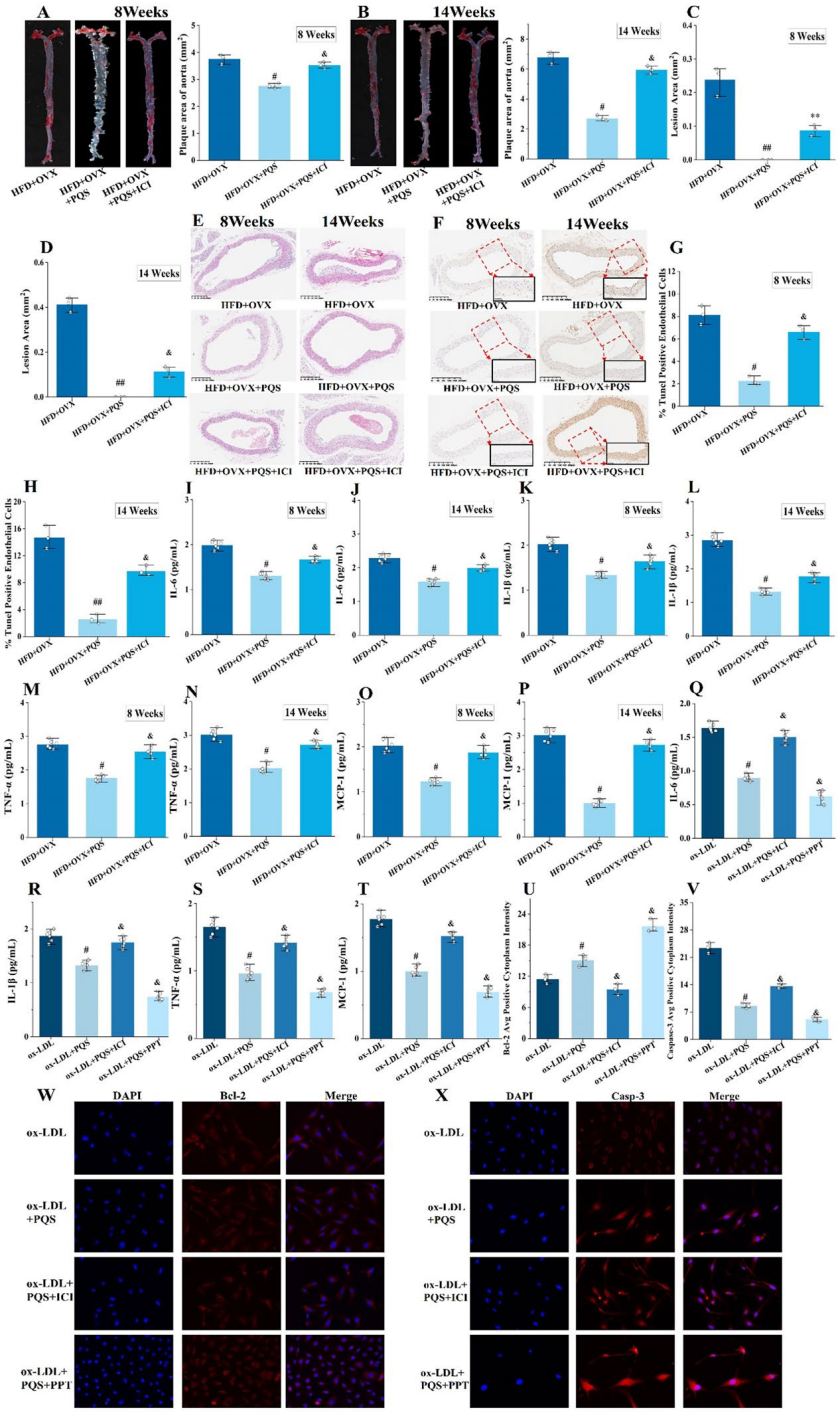
Inhibiting ERα counteracted the beneficial effects of PQS in vivo and vitro. **A**-**B** Oil red O staining of aorta and quantitative analysis of atherosclerotic plaque areas at 8 and 14 weeks. **C**–**E** H&E staining of aortic root and quantitative analysis of aortic root atherosclerotic plaque areas at 8 and 14 weeks. **F**–**H** Images and quantitative analysis of aortic TUNEL staining at 8 and 14 weeks. **I**–**P** The levels of serum IL-6, IL-1β, TNF-α and MCP-1 in HFD + OVX ApoE^−/−^ mice at 8 and 14 weeks. **Q**–**T** The levels of serum IL-6, IL-1β, TNF-α and MCP-1 in HUVECs co-treated with PQS and ox-LDL. **U**–**X** Immunofluorescence assays (200x) and quantitative analysis of Bcl-2 and caspase-3 in HUVECs co-treated with PQS and ox-LDL. n = 3–6. Data are expressed as the mean ± SEM. ^#^P < 0.05, ^##^P < 0.01, vs. HFD + OVX; ^&^P < 0.05 vs. HFD + OVX + PQS; ^#^P < 0.05 vs. ox-LDL; ^&^P < 0.05 vs. ox-LDL + PQS

In order to find the role of ERα in the effects of PQS for decreasing atherosclerosis, ERα inhibitor was applied in vivo and vitro. The results manifested that inhibiting ERα with ICI 182780 counteracted the effects of PQS on decreasing atherosclerotic plaque, apoptosis of endothelial cells, expression of inflammatory cytokines in ovariectomized ApoE^−/−^ mice at both 8 and 14 weeks (Fig. 6A–P). The vitro study also showed that inhibiting ERα with ICI 182780 counteracted the beneficial effects of PQS on apoptotic proteins (Bcl-2 and caspase-3) and expression of inflammatory cytokines (IL-6, IL-1β, TNF-α and MCP-1), while ERα activator PPT facilitated these effects (Fig. 6Q–X). These findings suggest that PQS may mitigate atherosclerosis in ovariectomized ApoE^−/−^ mice by regulating ERα and its associated PI3K/Akt pathway to curb inflammation, as well as the MEK/ERK1/2 pathway to prevent endothelial cell apoptosis.

## Discussion

In this study, we demonstrated that atherosclerotic plaques formed more obvious in ovariectomized ApoE^−/−^ mice compared with ApoE^−/−^ mice without ovariectomy. And PQS significantly decreased atherosclerotic plaque size in ovariectomized ApoE^−/−^ mice fed with HFD by decreasing inflammatory response and apoptosis of endothelial cells. The mechanism of PQS on atherogenesis in ovariectomized ApoE^−/−^ mice was associated with regulating ERα/PI3K/Akt and ERα/MEK/ERK1/2 pathways. These results will provide more robust theoretical support and practical insights for advancing PQS as a therapeutic target for atherosclerosis in postmenopausal women toward clinical application.

The prevalence of ACVD was higher in men than women until midlife. The decrease of estrogen levels during the period of menopause enhances the susceptibility of women to the risks of atherogenesis [10]. Our study revealed a remarkable decrease in serum estrogen levels in ovariectomized ApoE^−/−^ mice compared with those mice without ovariectomy. E2 treatment reduced the areas of atherosclerotic plaques at 8 weeks, but not at 14 weeks in ovariectomized ApoE^−/−^ mice, implying that HRT treatment should be considered at early stage of postmenopausal women. Previous clinical studies also demonstrated that HRT appears to be beneficial for women under the age of 60 compared with those over 60, particularly in reducing the risk of ACVD, osteoporosis, and cognitive impairment [8–11, 29]. In our study, we found that PQS treatment ameliorated the atherosclerotic plaque formation in en face aorta and aortic root of ovariectomized ApoE^−/−^ mice at both 8 and 14 weeks when compared with those mice without ovariectomy. Moreover, with the extended duration of PQS treatment, the beneficial effects of PQS on atherogenesis in ovariectomized ApoE^−/−^ mice fed with HFD became pronounced. Unlike HRT, which fails in long-term use, PQS sustains anti-atherogenic effects via reducing inflammatory response and apoptosis of endothelial cells. These findings indicate that PQS may be a probable novel therapy for atherogenesis in postmenopausal women.

Ovarian dysfunction could lead to the disorder in lipid metabolism, the increase of endothelial cells apoptosis and exacerbation of inflammatory response, which attribute to lipid deposition in aortic tissue and endothelial dysfunction [1, 30, 31]. Our study demonstrated that PQS treatment did not influence the lipids profile, indicating that the anti-atherosclerotic effects of PQS in ovariectomized ApoE^−/−^ mice may be associated with other mechanism. Our findings showed that PQS treatment decreased inflammatory response and endothelial cells apoptosis in ovariectomized ApoE^−/−^ mice at both 8 and 14 weeks, and more pronounced effects of PQS were observed at 14 weeks. These results demonstrate that PQS protecting against atherogenesis in ovariectomized ApoE^−/−^ mice might be attributed to decrease inflammatory response and endothelial cells apoptosis.

To explore the possible mechanism by which PQS decreased atherosclerotic plaque size in ovariectomized ApoE^−/−^ mice, the network pharmacology and molecular docking analysis were performed, and the results showed that PQS decreased atherosclerotic plaque size in ovariectomized ApoE^−/−^ mice by regulating ERα/PI3K/Akt and ERα/MEK/ERK1/2 signaling pathways. ERα, a nuclear receptor belonging to the ER family, is primarily expressed in breast, uterus, ovaries, and other reproductive organs of humans and other mammals. ERα is a key regulatory factor in estrogen signaling pathway involving in regulating cell survival, proliferation, and differentiation [2]. During the development of atherosclerosis in menopause women, endothelial dysfunction is associated with endothelial cells apoptosis, and involved in reduction of estrogen and ERα [32]. Estrogens have great benefits to exert anti-inflammatory effects by binding with ERα. Aging, estrogen loss and ERα reduction are indelibly linked in menopausal women [33]. Aging is connected with inflammatory response and raised inflammatory cytokines expression, such as TNF-α and IL-6, thus impairing endothelial function [33]. Our findings show that ERα expression was reduced in ovariectomized ApoE^−/−^ mice fed with HFD and ox-LDL-treated HUVECs, whereas it was significantly increased by PQS treatment. Inhibiting ERα with ICI 182780 counteracted the effects of PQS on decreasing atherosclerotic plaque in ovariectomized ApoE^−/−^ mice fed with HFD at both 8 and 14 weeks.

PI3K/Akt is downstream pathways of ERα, inactivation of ERα decreased PI3K and Akt expression, which accelerated apoptosis of endothelial cells by inducing LDH release, impairing mitochondrial potential, raising Bax levels relative to Bcl-2, and stimulating caspase-3 cleavage, thus facilitating atherogenesis [34, 35]. Our results exhibited that PI3K and p-Akt expression were lowered in both ovariectomized ApoE^−/−^ mice fed with HFD and ox-LDL-treated HUVECs, while PQS treatment increased PI3K and p-Akt. Both ERα activator and inhibitor were employed in injured HUVECs induced by ox-LDL, and these findings displayed that the effects of PQS on activating PI3K and p-Akt were enhanced following ERα activation, while ERα inhibition mostly offset these effects. Moreover, PQS treatment facilitated Bcl-2 expression and lowered caspase-3 expression in vivo and vitro, while ERα inhibitor offset these effects. These findings indicate that the mechanism of PQS on decreasing atherogenesis in ovariectomized ApoE^−/−^ mice may be associated with the ERα/PI3K/Akt pathway, as PQS treatment increased the expression of p-Akt (Ser473) and total PI3K. The anti-apoptotic effect was further supported by decreased TUNEL-positive cells, increased Bcl-2, and decreased pro-caspase-3 levels. MEK/ERK1/2 signals are downstream pathways of ERα, and their activation is closely associated with inflammatory response by increasing expression of pro-inflammatory cytokines. Our results manifested that MEK and ERK1/2 expression were enhanced in both ovariectomized ApoE^−/−^ mice fed with HFD and ox-LDL-treated HUVECs, while PQS treatment decreased MEK and ERK1/2. The vitro study revealed that the effects of PQS on inactivating MEK and ERK1/2 were enhanced following ERα activation, while ERα inhibition mostly offset these effects. Moreover, PQS treatment decreased the levels of serum IL-6, IL-1β, TNF-α and MCP-1, and NF-κB expression in vivo and vitro, while ERα inhibitor offset these effects. These findings indicate that the mechanism of PQS on decreasing atherogenesis in ovariectomized ApoE^−/−^ mice may involve the ERα/MEK/ERK1/2 pathway, as PQS treatment reduced the total protein expression of MEK and ERK1/2. This effect, along with the reduced p-p65/p65 ratio and inflammatory cytokine levels, suggests a potential link to the inflammatory response. This is achieved through suppression of pro-inflammatory cytokine production, interruption of critical inflammatory cascades, and facilitation of resolution mechanisms. Notably, inflammation can induce vascular hyperplasia independently of conventional cardiovascular risk factors and influences plaque biology, thereby exacerbating advanced atherosclerotic complications. Consequently, targeting inflammation may also mitigate lipid accumulation.

Furthermore, it is crucial to consider the synergistic interplay between the ERα/PI3K/Akt and ERα/MEK/ERK1/2 pathways in mediating the protective effects of PQS. The PI3K/Akt pathway is predominantly associated with promoting cell survival and inhibiting apoptosis [34, 35], while the MEK/ERK1/2 pathway is more directly linked to the regulation of inflammatory responses [36]. However, these pathways do not function in isolation. There is extensive crosstalk between these two signaling cascades. For instance, Akt can cross-regulate components of the ERK pathway, and both pathways can converge on shared downstream effectors, such as NF-κB and Bcl-2 family proteins, to jointly determine the cellular fate between survival and death [37, 38]. Our results demonstrate that PQS, via the upstream activation of ERα, simultaneously modulates both pathways: enhancing the anti-apoptotic PI3K/Akt signal while suppressing the pro-inflammatory MEK/ERK1/2 axis. This dual regulation creates a synergistic protective network, wherein reduced inflammation provides a more favorable microenvironment for endothelial cell survival, and enhanced cell survival, in turn, contributes to the maintenance of vascular integrity and function. This coordinated mechanism likely underlies the pronounced and sustained anti-atherogenic effect of PQS observed in our study.

Our previous studies have demonstrated the efficacy and safety of PQS in treating patients with CHD [20, 39]. Specifically, our clinical trial [20] indicated that PQS, when used as an adjunctive therapy, did not induce significant adverse effects on liver, renal, or coagulation functions during the treatment period, suggesting a favorable safety profile for clinical use. Although long-term safety data in postmenopausal populations specifically are still being accumulated, the extensive traditional use and existing clinical evidence provide a strong foundation for its tolerability. Moreover, considering the protective effect of PQS on atherogenesis in ovariectomized ApoE^−/−^ mice fed with HFD, potential therapeutic applications of PQS could be explored, such as its viability as an adjunct therapy for postmenopausal women with high-risk atherosclerosis, particularly those are refractory to HRT. The mechanistic insights gained from this study, revealing its multi-targeted action through ERα-mediated pathways with a favorable safety baseline, significantly strengthen the rationale for its further clinical translation.

In current experiment, we employed a model of atherosclerosis in ovariectomized ApoE^−/−^ mice with feeding HFD, which can’t completely replicate the pathogenic process in postmenopausal women, because there is no established method to directly mimic atherogenesis relative to menopause to date. Adding histopathological characterization at different pathological stages would strengthen the changes of the pathogenic process. In addition, since ApoE^−/−^ mice fed a HFD can also develop atherosclerosis, the effects of PQS on ApoE^−/−^ mice with or without ovariectomy may not be entirely distinguishable. However, we found that more pronounced atherosclerotic plaques were observed in ovariectomized ApoE^−/−^ mice compared with ApoE^−/−^ mice without ovariectomy, while PQS treatment decreased atherosclerotic plaque size in ovariectomized ApoE^−/−^ mice. Simultaneously, ERα inhibitor offset the protective function of PQS on atherogenesis, suggesting that the effects of PQS on atherosclerosis in ovariectomized ApoE^−/−^ mice were correlative with regulating ERα. Although we assessed pro-caspase-3 expression rather than its cleaved/activated form, the observed reduction in pro-caspase-3 was consistent with decreased apoptosis by TUNEL and flow cytometry and increased Bcl-2 expression. Future studies using cleaved caspase-3-specific antibodies to quantify the cleaved/pro-caspase-3 ratio would provide more direct evidence of caspase-3 activation and further strengthen our mechanistic conclusions. It should be noted that while our study demonstrates changes in total protein expression of PI3K, MEK, and ERK1/2, the assessment of their phosphorylated forms would provide more direct evidence of pathway activation. Future studies incorporating phospho-specific antibodies are warranted to further elucidate the precise mechanism by which PQS modulates these signaling cascades.

## Conclusion

PQS decreased atherogenesis in ovariectomized ApoE^−/−^ mice fed with HFD by decreasing inflammatory response and apoptosis of endothelial cells. The underlying mechanism was associated with altered protein expression in the ERα/PI3K/Akt and ERα/MEK/ERK1/2 pathways, including increased p-Akt and decreased total MEK/ERK1/2. However, further studies with phosphorylation-specific antibodies are required to confirm the activation status of these pathways. Therefore, our findings suggested that PQS would be a promising complementary botanic medicine for atherosclerosis in postmenopausal women.

## Supplementary Information

Supplementary material 1.

Supplementary material 2.

Supplementary material 3.

Supplementary material 4.

Supplementary material 5.

## Data Availability

The data that support the findings of this study are available from the corresponding author upon reasonable request.

## Abbreviations

CHD: Coronary heart disease
ERα: Estrogen receptor alpha
FITC: Fluorescein isothiocyanate
HDL-C: High-density lipoprotein cholesterol
H&E: Hematoxylin and eosin
HFD: High-fat diet
HPLC–UV: High-performance liquid chromatography ultraviolet
HRT: Hormone replacement therapy
HUVECs: Human umbilical vein endothelial cells
ICI: ICI 182780
LDL-C: Low-density lipoprotein cholesterol
OVX: Ovariectomized
ox-LDL: Oxidized low-density lipoprotein
PQS: Panax quinquefolium saponin
PPT: Propyl pyrazole triol
SMCs: Smooth muscle cells
TC: Cholesterol
TG: Triglyceride
TCM: Traditional Chinese medicine
TUNEL: TdT-mediated dUTP nick-end labeling
WHI: Women's Health Initiative
