# Supplementary material for: Panax quinquefolium saponin decreases atherosclerosis in ovariectomized ApoE−/− mice via regulating estrogen receptor α
Source: Chin Med. 2026 Jun 22;21:171. doi: 10.1186/s13020-026-01410-3 (PMC13285194; doi:10.1186/s13020-026-01410-3)
Supplement: Supplementary file 1 — Supplementary material 1. [file 13020_2026_1410_MOESM1_ESM.docx]

Table S1 Primer sequences for RT-PCR.

| Primer sequences  ERα Forward primer  ERα Reverse primer  PI3K Forward primer  PI3K Reverse primer  Akt Forward primer  Akt Reverse primer  Bcl2 Forward primer  Bcl2 Reverse primer  Caspase3 Forward primer  Caspase3 Reverse primer  ERK Forward primer  ERK Reverse primer  MEK Forward primer  MEK Reverse primer  NF-κB Forward primer  NF-κB Reverse primer  GAPDH Forward primer  GAPDH Reverse primer | Primer  5'-AAGACGCTCTTGAACCAGCA-3’  5'-AGGCTTTGGTGTGAAGGGTC-3’  5'-TCCAAATACCAGCAGGATCA-3’  5'-ATGCTTCGATAGCCGTTCTT-3’  5'-GAACGGCCTCAGGATGTGGA-3’  5'-GGTGCGCTCAATGACTGTGG-3’  5'-GCATGCGACCTCTGTTTGAT-3’  5'-CAGGTATGCACCCAGAGTGA-3’  5'-GAGCTTGGAACGGTACGCTA-3’  5'-GAGTCCACTGACTTGCTCCC-3’  5'-TTCCAAGGGTTATACCAAGTCC-3’  5'-TCAGCTGGTCAAGGTAATGC-3’  5'-GGTGGAGTGGTCTTCAAGGT-3’  5'-GGTGGATCAGCTTTCTAGCC-3’  5'-GCTACACAGAGGCCATTGAA-3’  5'-TCCCGGAGTTCATCTATGTG-3’  5'-TGCCCCCATGTTTGTGATG-3’  5'-TGTGGTCATGAGCCCTTCC-3’ | Products  121 bp  137 bp  144 bp  95 bp  118 bp  110 bp  67 bp  142 bp  151 bp |
| --- | --- | --- |
