# Supplementary material for: Panax quinquefolium saponin decreases atherosclerosis in ovariectomized ApoE−/− mice via regulating estrogen receptor α
Source: Chin Med. 2026 Jun 22;21:171. doi: 10.1186/s13020-026-01410-3 (PMC13285194; doi:10.1186/s13020-026-01410-3)
Supplement: Supplementary file 2 — Supplementary material 2. [file 13020_2026_1410_MOESM2_ESM.docx]

Table S2 Binding affinities and predicted inhibition constants (pKi) of molecular docking.

| Protein | estrogen receptor α | |
| --- | --- | --- |
| Compounds | Binding affinity (kcal/mol) | pKi (μM) |
| Ginsenoside Re | -6.83±0.16 | 5.28±0.00 |
| Ginsenoside Rb2 | -6.83±0.40 | 12.39±2.08 |
| Ginsenoside Rb1 | -6.63±0.29 | 14.28±7.95 |
| Ginsenoside Rc | -8.20±0.52 | 7.09±1.50 |
| Ginsenoside Rg1 | -6.53±0.06 | 13.78±1.30 |
