# Supplementary figures and images for: Panax quinquefolium saponin decreases atherosclerosis in ovariectomized ApoE−/− mice via regulating estrogen receptor α
Source: Chin Med. 2026 Jun 22;21:171. doi: 10.1186/s13020-026-01410-3 (PMC13285194; doi:10.1186/s13020-026-01410-3)

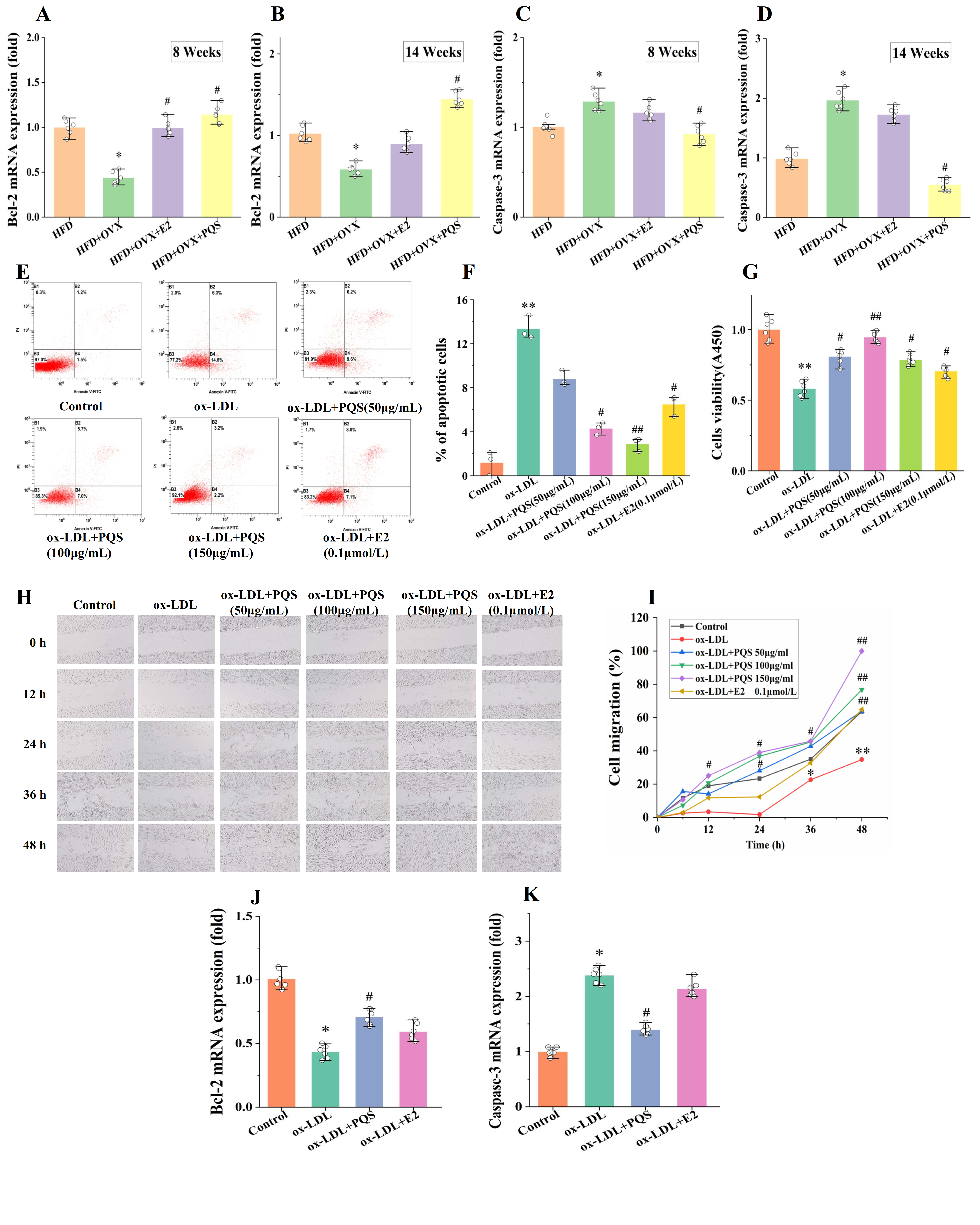

Supplement: Supplementary file 3 — Supplementary material 3. [file 13020_2026_1410_MOESM3_ESM.tif]

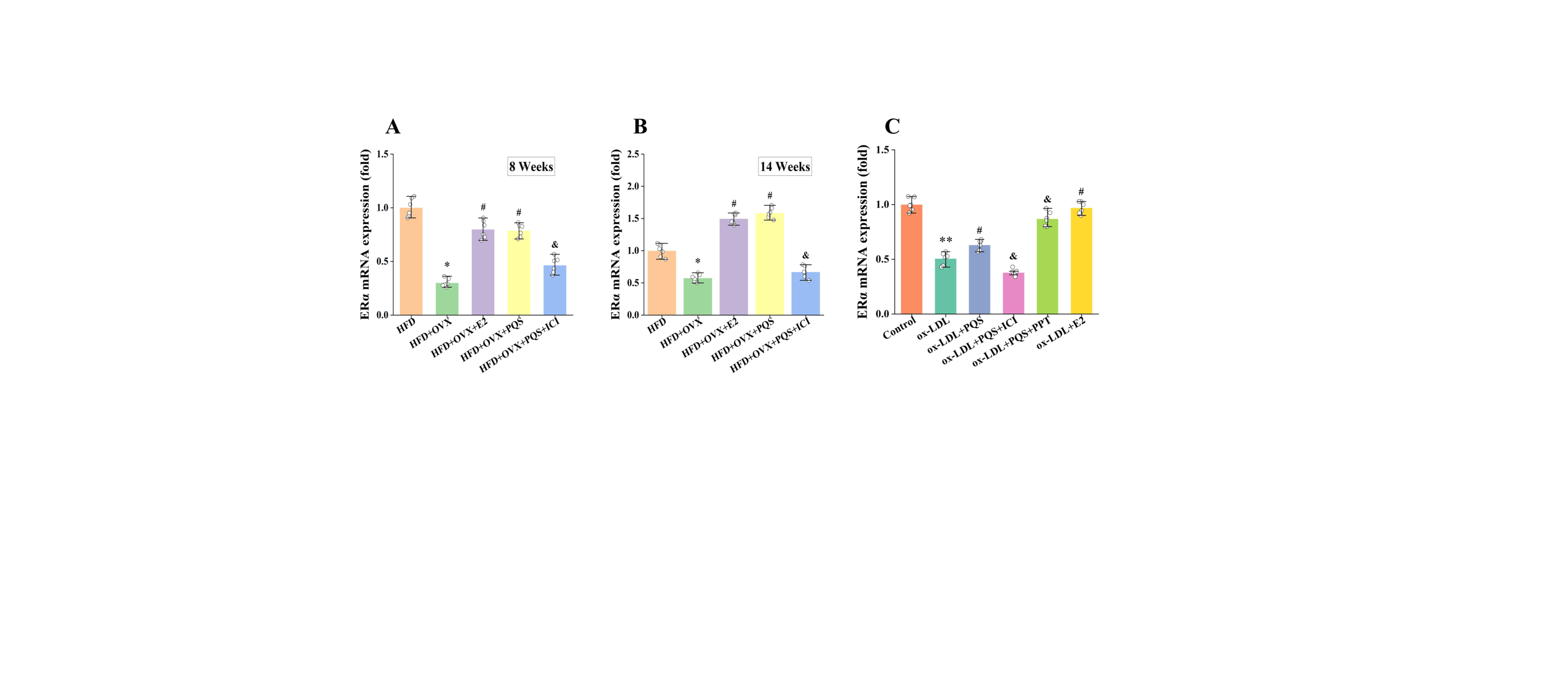

Supplement: Supplementary file 4 — Supplementary material 4. [file 13020_2026_1410_MOESM4_ESM.tif]

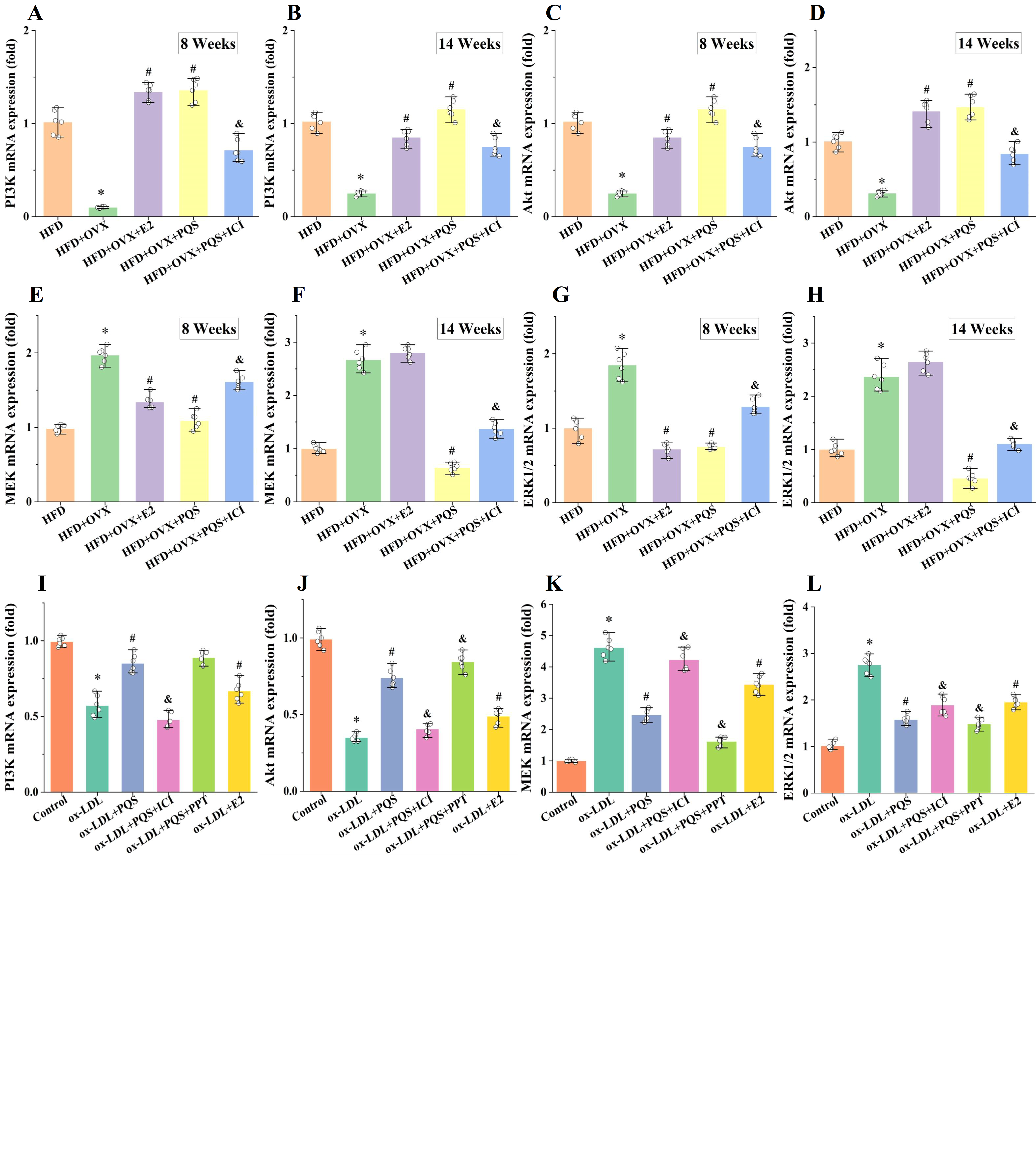

Supplement: Supplementary file 5 — Supplementary material 5. [file 13020_2026_1410_MOESM5_ESM.tif]
